# Lymphatic channel sheet of polydimethylsiloxane for preventing secondary lymphedema in the rat upper limb model

**DOI:** 10.1002/btm2.10371

**Published:** 2022-07-05

**Authors:** Hwayeong Cheon, Ma. Nessa Gelvosa, Sang Ah Kim, Ho‐Young Song, Jae Yong Jeon

**Affiliations:** ^1^ Biomedical Engineering Research Center Asan Institute for Life Sciences, Asan Medical Center Seoul Republic of Korea; ^2^ Department of Rehabilitation Medicine Asan Medical Center, University of Ulsan College of Medicine Seoul Republic of Korea; ^3^ Department of Minimal‐Invasive Intervention The Affiliated Cancer Hospital of Zhengzhou University Zhengzhou City China

**Keywords:** artificial lymphatic pathway, lymphangiogenesis, lymphatic channel sheet, polydimethylsiloxane, secondary lymphedema

## Abstract

Secondary lymphedema is a severe complication of cancer treatment, but there is no effective curative method yet. Lymph node dissection and radiation therapy for cancer treatment may lead to secondary lymphedema, which is a chronic disease induced by malfunction of lymphatic flow. The lymphatic channel sheet (LCS) is an artificial micro‐fluidic structure that was fabricated with polydimethylsiloxane to maintain lymphatic flow and induce lymphangiogenesis. The structure has two‐dimensional multichannels that increase the probability of lymphangiogenesis and allow for relatively easy application. We verified the efficacy of the lymphatic channel sheet through macroscopic and microscopic observation in small animal models, which underwent brachial lymph node dissection and irradiation. The lymphatic channel sheet enabled the successful transport of lymphatic fluid from the distal to the proximal area in place of the removed brachial lymph nodes. It prevented swelling and abnormal lymphatic drainage during the follow‐up period. Lymphangiogenesis was also identified inside the channel by histological analysis after 8 weeks. According to these experimental results, we attest to the roles of the lymphatic channel sheet as a lymphatic pathway and scaffold in the rat upper limb model of secondary lymphedema. The lymphatic channel sheet maintained lymphatic flow after lymph node dissection and irradiation in an environment where lymph flow is cut off. It also relieved symptoms of secondary lymphedema by providing a lymph‐friendly space and inducing lymphangiogenesis.

## 1 INTRODUCTION

Secondary lymphedema is a lymphatic disease caused by an acquired defect in the lymphatic system. The damaged lymphatic circulation occurs due to the imbalance between production and drainage in the lymphatic system leading to swelling, which is the accumulation of extracellular fluid, proteins, and cell debris in the interstitial tissue.^1^, ^2^, ^3^, ^4^ Lymphedema refers to the irreversible progress of abnormal lymphatic circulation that consequently induces the swelling of the extremities, worsening fibrosis, recurrent cellulitis, and excessive adipose tissue deposition. These further disrupt lymphatic transport such as fibrosis, infection, and adipocyte deposition creating a vicious cycle that can lead to aggravation of lymphedema symptoms chronically.^5^, ^6^, ^7^, ^8^ The damage of lymphatics can result from various causes such as cancer treatment, injury trauma, venous disease, obesity, inflammation, skin infection, drug abuse, or infection of parasites, and so on, but the cancer treatments including the surgical process, radiotherapy, and chemotherapy are the leading causes.^9^

The surgical procedure, particularly lymph node dissection, physically disconnects the lymphatic pathway because lymphatic vessels and lymph nodes serve as a draining channel for returning lymphatic fluid from interstitial tissue spaces to the venous system.^1^, ^10^, ^11^ The absence or removal of lymph nodes is like losing a major drainage pathway since lymph nodes are located at strategic positions along with the lymphatic system. In the case of upper limbs, the bulk of the lymphatic load is gathered in the axillary lymph nodes (ALNs) located in the lymphatic plexus bundles along with intersecting nerves, blood vessels, and lymphatic vessels. Because of its location, axillary lymph node dissection (ALND) can lead to the disruption of the lymphatic system in the upper limbs.^1^, ^6^, ^12^ The occurrence rate of breast cancer‐related lymphedema varies according to the reported research, but it is generally expected 20%–50% after ALND, and 5%–20% after sentinel lymph node dissection.^1^, ^13^, ^14^, ^15^, ^16^, ^17^, ^18^ While the surgery may have an immediate and critical effect on the lymphatic system, radiotherapy in the plexus area affect lymphatic flow extensively over the long term. The short‐term effect of radiation on the structure of lymphatic vessels is limited, but radiation depletes lymphatic endothelial cells (LECs) by the inhibition of lymphatic growth factors and induces fibrosis of soft tissue and lymph nodes.^4^, ^19^ This long‐term alteration can develop dysfunction of the lymphatic system,^12^ and a combination of ALND and radiation therapy may have synergistic effects, which increase the risk of lymphedema.^12^, ^20^

The recovery of lymphatic transport capability is a key factor in the treatment of lymphedema. Manual lymphatic drainage (MLD) has been widely used as a gold standard of conservative treatment because MLD of complex decongestive therapy increases the lymphatic transport temporarily. However, the goal of this strategy is not recovery of damaged lymphatic pathways and it offers a temporary improvement with low effectiveness in some patients such as those with lymphedema of lower extremities or persistent edema.^21^, ^22^, ^23^ Another strategy is a surgical intervention to create a bypass of lymphatic flow or induce lymphangiogenesis.^21^, ^24^, ^25^ The surgical treatments including lymphovenous anastomosis (LVA) and vascularized lymph node transfer (VLNT) aim to rebuild the lymphatic pathway. LVA creates a bypass that is connected to the venous system and allows the reduction of residual lymphatic fluid.^26^, ^27^ Because it is necessary to know accurate information about the patient's lymphatic vessels for LVA, the success rate of anastomosis is greatly affected by the condition of the recipient site.^28^ VLNT, which is another microsurgical technique, restores physiological lymphatic flow in lymphedema extremities by transferring functional lymph nodes and expecting lymphangiogenesis from the transferred lymph node.^29^, ^30^, ^31^, ^32^ Although lymphangiogenesis in VLNT, which is a key treatment mechanism, has the advantage of reducing the limitation of the recipient site, there is a risk of donor site complications.^33^ On the other hand, Yamamoto et al. also suggested other surgical interventions for creating a new lymphatic pathway by subdermal dissection recently. They demonstrated subdermal dissection to create the pathway for inducing neo‐lymphangiogenesis, but it has the limitation in the lack of histological studies to confirm lymphangiogenesis to validate their results.^34^ These surgical interventions restore the lymphatic pathway directly, but they are effective mostly in the early stages of lymphedema and there is a poor prognosis for extensive operative scars, irradiated tissue, or serious fibrotic tissues.^35^

To overcome the limitation of the current strategy, an artificial lymphatic pathway can be a new strategy for the effective treatment of lymphedema. The advantage of the artificial lymphatic pathway is that it may be applied to patients of all stages relatively easily compared to conventional surgical intervention without requiring a donor. The goal of the artificial implant structure is the transfer of stagnant lymphatic fluid into the proximal area across the fibrotic tissues and to provide a conducive environment for lymphangiogenesis, but existing artificial lymphatic structures with tubular forms have been inefficient in both lymphangiogenesis and lymphatic transport. Here, we fabricated two‐dimensional sheet‐type lymphatic channels (lymphatic channel sheet [LCS]) with polydimethylsiloxane (PDMS) and verified its effect in the prevention of secondary lymphedema in a rat upper limb model in which the lymph nodes were dissected. We examined the role of LCS as a pathway to reconnect the damaged lymphatic vessels and its role as a scaffold that allows the formation of new lymphatic vessels.

## 2 RESULTS

### 2.1 Reconnection of lymphatic flow by the LCS

**FIGURE 1:**
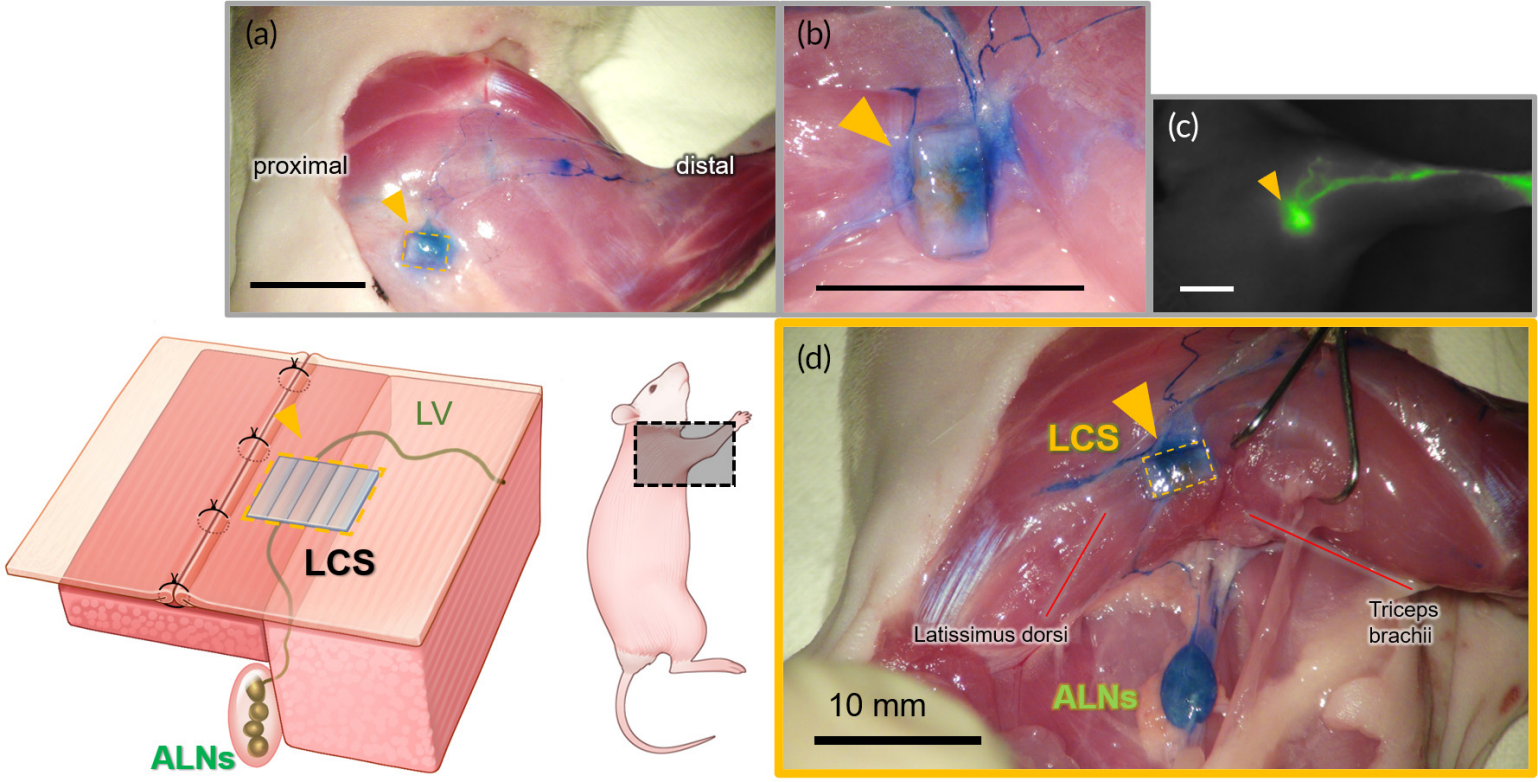
After 8 weeks of implantation, (a) the lymphatic flow through the lymphatic channel sheet (LCS) (yellow triangle) of polydimethylsiloxane (PDMS) from the distal to the proximal area could be identified by the Evans blue (EB) dye. (b) It is seen that the lymphatic vessels were connected at both ends of the LCS after removing the surrounding tissue in the magnified view. (c) The indocyanine green (ICG) fluorescence image of the lymphatic flow near the LCS. The lymphatic fluid was collected from the LCS. (d) The anatomical detail of the lymphatic flow from nearby the LCS to axillary lymph nodes (ALNs). The EB dye, which was injected into the palm, flowed into ALNs throughout the LCS. The pectoralis major was incised to identify lymph vessels from the distal area to ALNs and surrounding the LCS. All figures were from the same animal and all scale bars are 10 mm.

**FIGURE 2:**
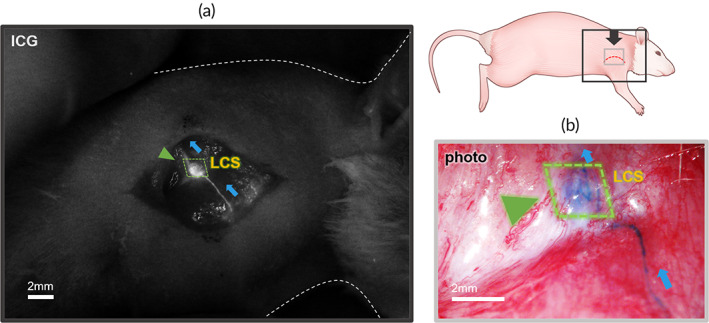
Visualized lymphatic flow surrounding the lymphatic channel sheet (LCS) by (a) indocyanine green (ICG) fluorescence dye injection. The ICG was infused into the LCS and the structure of the LCS was revealed (green triangle). (b) The Evans blue (EB) dye injection stained the channels inside the LCS (green triangle). The images of ICG and EB injection were obtained from the dorsal position of the same animal. All scale bars are 2 mm.

No apparent inflammation in the implantation area of the LCS limb was observed during the follow‐up period. The lymph node dissection and irradiation procedure in the proximal area blocked the lymphatic transport in the resection limb. Nevertheless, the LCS maintained the lymphatic flow from the distal area of the upper limbs to the proximal lymph nodes (ALN) in the LCS limbs of the animal models. Figure 1 shows the implanted LCS after 8 weeks, and the lymphatic vessels were connected to both edges of the LCS. Lymphatic flow, which was visualized by Evans blue (EB) dye injected in the distal paw, passed through the LCS and reached into the ALN. The detailed connection between lymphatic vessels and LCS was observed using microscopy for the near‐infrared and visible spectrum. As shown in Figure 2, the lymphatic fluid was transported along the existing lymphatic vessels and the LCS structure in both the indocyanine green (ICG) fluorescence image and EB dye image. Because of these results, it has been confirmed macroscopically that the LCS was being used as the major pathway from the distal area to the proximal area.

### 2.2 Investigation of the indicator

**FIGURE 3:**
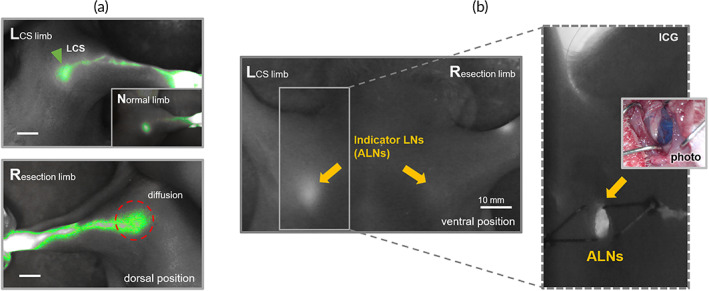
(a) Indocyanine green (ICG) pattern for lymphatic drainage after 8 weeks of implantation of the lymphatic channel sheet (LCS). The lymphatic flow in the LCS limb was concentrated in the lymphatic vessels and collected into the LCS. This pattern was almost the same with the normal limb (inset figure; the lymphatic flow was collected linearly into BLNs in the normal limb). (b) ICG image of the indicator (yellow arrows) in both LCS limb and resection limb. The bright spot in the indicator LN represented that lymphatic flow including the ICG dye was drained into the axillary lymph nodes (ALNs) (the image with the dotted line and inset image). All scale bars are 10 mm.

We observed the visualization of the ALNs as the indicator and ICG pattern of dorsal upper limbs every week after the surgical procedure and irradiation. The indicators were identified clearly in all of the LCS limbs during the follow‐up period while they looked blurry or faint in the resection limbs (Figure S1). We selected individuals with poor lymph node dissection through observation of indicators and excluded them from the measurement (*n* = 1). Figure 3 presents the flow pattern of lymphatic fluid and the status of the indicator according to it in both limbs. In the LCS limbs, the flow pattern was mainly concentrated in lymphatic vessels and the LCS, and it was almost the same as that of BLNs in a normal limb. The brighter visible indicator and the flow pattern indicated that lymphatic fluid was effectively flowing to the indicator of the proximal area through the LCS. However, it was observed that the flow of lymphatic fluid was not smooth in the resection limbs. There was a diffusion of lymphatic fluid to the periphery of the lymphatic vessels and lymph node dissection area. The indicator was barely visible on the side of the resection limbs because lymph node dissection and irradiation blocked lymphatic flow from the distal area.

**FIGURE 4:**
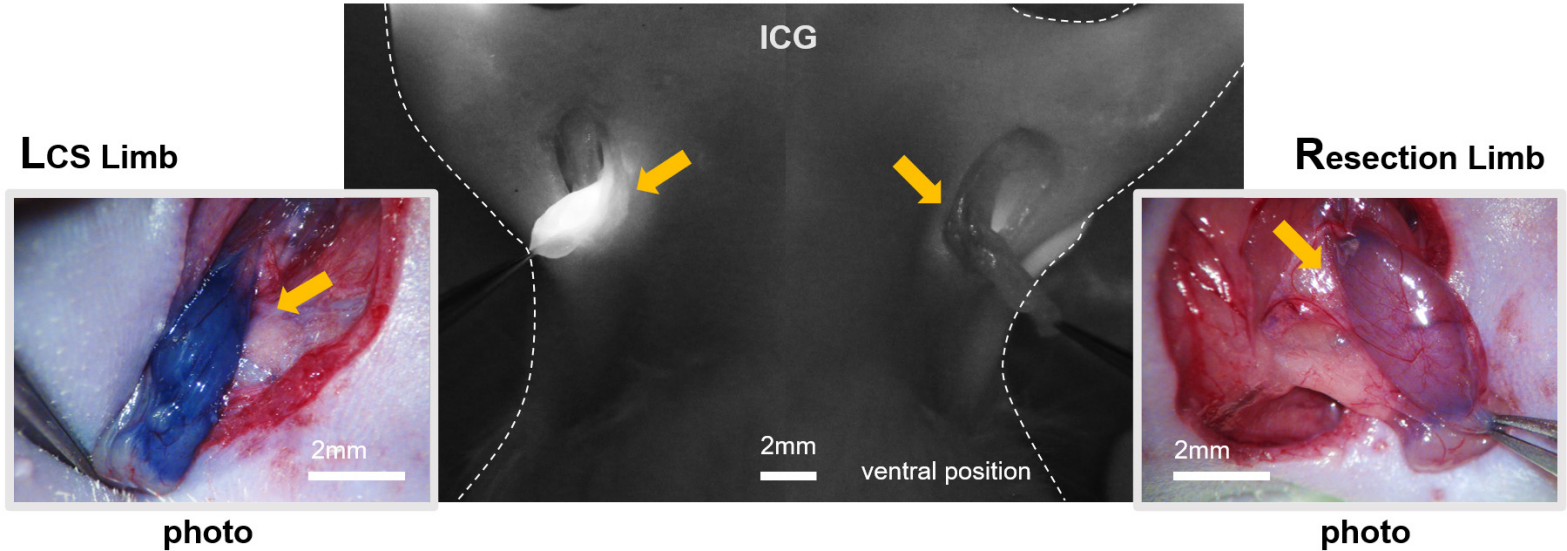
The exposed indicator or axillary lymph nodes (ALNs) (yellow arrows) in both lymphatic channel sheet (LCS) limb and resection limb after injection of indocyanine green (ICG) fluorescent and Evans blue (EB) dye. In the LCS limbs, we could detect both dyes almost immediately in the indicator after injection. At 30 min after injection, the dyes were rarely observed in the resection limb, although a few were detected. All scale bars are 2 mm.

To investigate the condition of the indicator in detail, we exposed the indicator by axillary skin incision (Figure 4). The EB dye and ICG fluorescent dye injected in the distal area were visualized in the indicator (ALNs) of the LCS limbs, whereas both dyes could hardly be detected in ALNs of the resection limbs.

### 2.3 ICG pattern of lymphatic drainage

**FIGURE 5:**
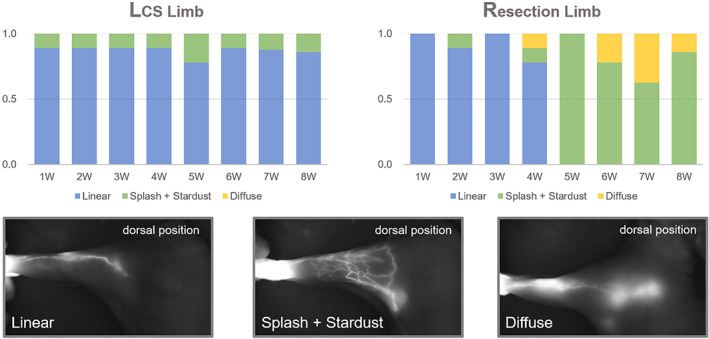
The indocyanine green (ICG) pattern change of dorsal position in both lymphatic channel sheet (LCS) and resection limb. The patterns were observed every week for 8 weeks. They were classified based on the images below the graph, and the graph presents the normalized numerical values for the frequency of each pattern because the patterns may appear in combination. In the LCS limb, the linear pattern was maintained for 8 weeks, whereas in resection, it gradually changed to an abnormal lymph flow pattern (splash, stardust, diffuse) from 5 weeks. These results were averaged by an observer measuring three times each.

The lymphatic pattern in indocyanine green (ICG) lymphangiography (ICG pattern) represents the condition of lymph flow and the dermal backflow and abnormal lymphatic transport can be identified from it. The dermal backflow was classified into the following ICG patterns in increasing severity: splash, stardust, and diffuse pattern. Since there can be several patterns on one limb, the pattern occupying the widest area was observed and counted for 8 weeks. We excluded inaccurate individuals from the measurement due to poor lymph node dissection (*n* = 1). As the result, the majority of the LCS limbs maintained the normal linear pattern throughout the follow‐up period. On the other hand, although the resection limbs exhibited the linear pattern initially, all of them converted to either splash or stardust patterns on the fifth week and later to diffuse patterns on the sixth until the eight week (Figure 5). These results were obtained by averaging three measurements.

### 2.4 Volume measurement

**FIGURE 6:**
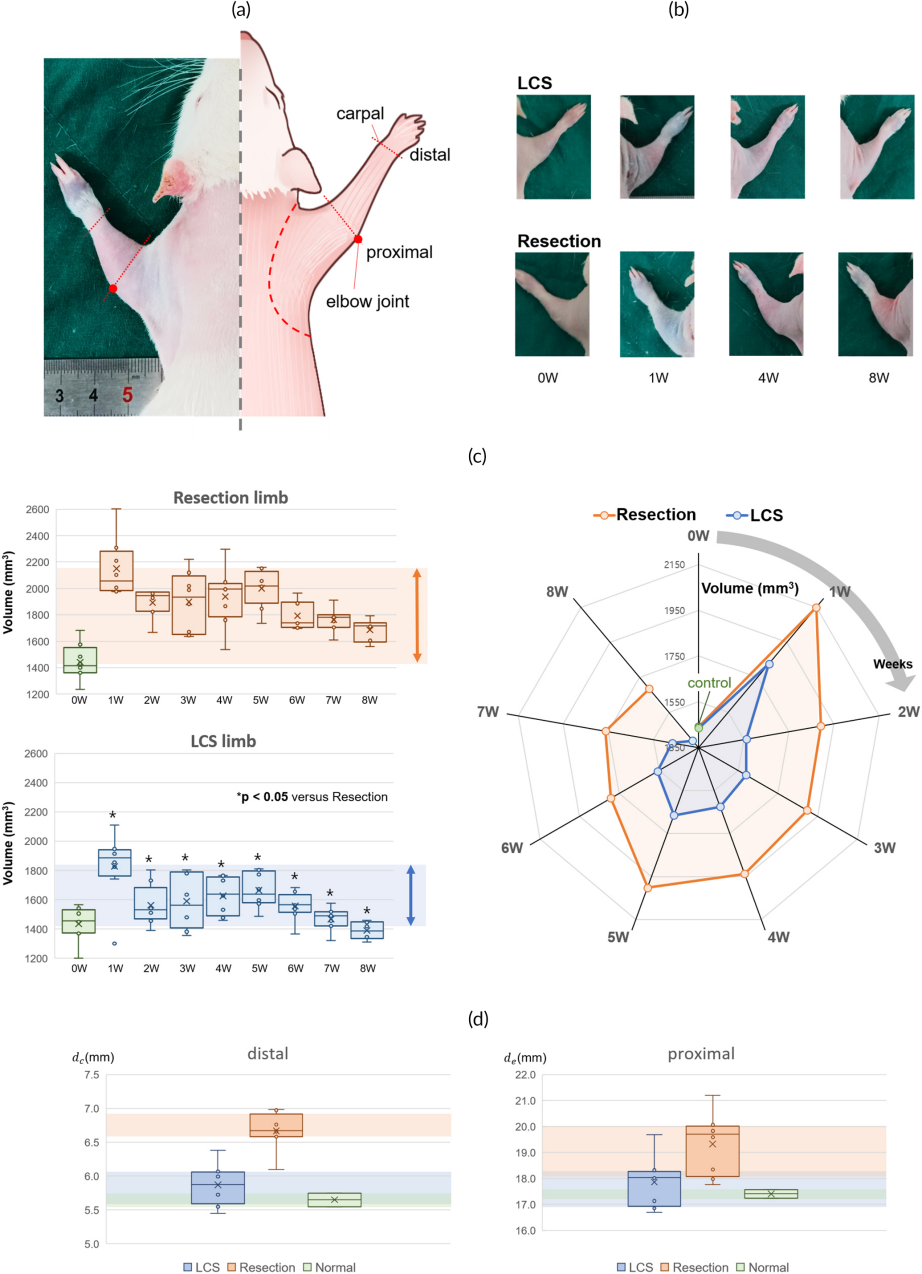
(a) The anatomical reference for volume measurement and (b) the images of each limb to measure the diameter using the ImageJ software. (c) The change of the volume in both lymphatic channel sheet (LCS) and resection limb for 8 weeks. The colored background presents the fluctuation range of the volume change. There was a significant difference between the LCS and resection limb in all follow‐up periods. *p < 0.05 versus the volume of resection limb based on ANOVA. The radar graph presents the difference between the volume of the LCS and resection limb each week. The LCS limb returned to near normal values in the eighth week. (d) The box and‐whisker diagram of diameter in the distal area (carpel) and proximal area (from elbow to cubital fossa) during 8 weeks. The resection limb had a significant difference compared to the normal limb, but the difference in the distal area was more pronounced. There was no significant difference in the LCS limb compared to the normal limb. The colored background presents from Q1 (the median of the lower half of the dataset) to Q3 (the median of the upper half of the dataset) in the box plot.

The volume of the upper limb from the carpal to the elbow joint was measured every week during the follow‐up period (Figure 6a,b). We also excluded inaccurate individuals from the measurement due to poor lymph node dissection as with the ICG pattern observation (*n* = 1). The volume of both the LCS and resection limbs averaged approximately 1450 mm^3^ before the surgical/radiation procedure. In the first week, the volume in both limbs was increased rapidly due to surgical trauma, but the resection limbs showed a larger increase of about 400 mm^3^ than that of the LCS limb (*p* > 0.05). Edema in the LCS limb significantly reduced after the second week and the fluctuation range of volume change in the LCS limb remained lower than that in the resection limb throughout the whole follow‐up period (resection limb < 750 mm^3^, LCS limb < 400 mm^3^, *p* < 0.05). The reduction of swelling was observed after 6 weeks in both limbs, and the LCS limbs were back to normal size after the seventh week though edema was maintained in the resection limb (Figure 6c). The significant difference in volume between both limbs was derived from the difference in the distal area. Figure 6d presents the averaged diameter of distal ($d_{c}$) and proximal ($d_{e}$) measurements. The interquartile range (IQR) of the resection limbs (0.34) was smaller than that of the LCS limbs (0.47) in the distal area while the IQR of the resection limbs (1.95) was larger than that of the LCS limbs (1.35) in the proximal area. There was a significant difference in both the distal and proximal area between the resection and normal limb (*p* < 0.05), but the LCS limb did not have the difference as the normal limb.

### 2.5 Lymph flow dependence and lymphangiogenesis

**FIGURE 7:**
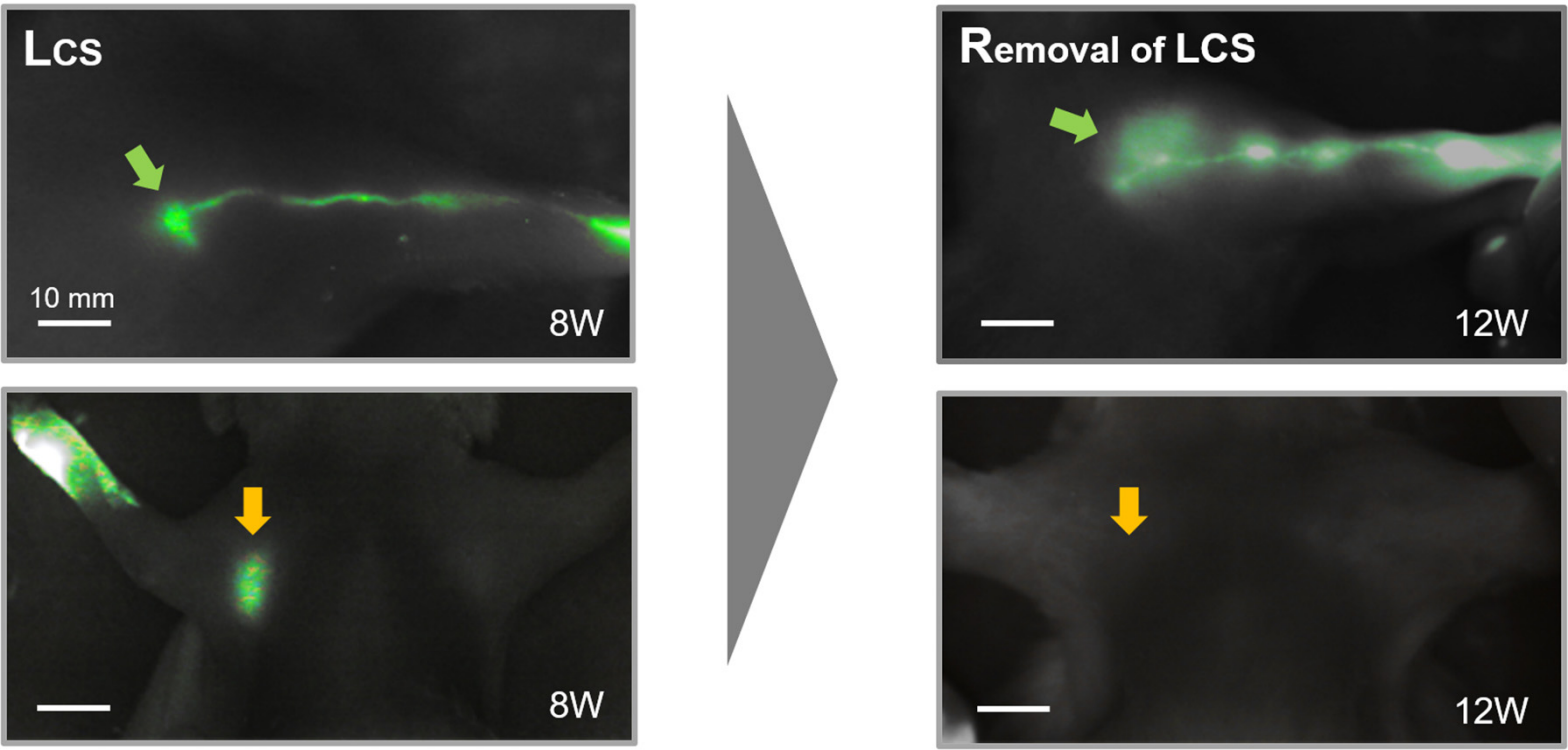
Indocyanine green (ICG) fluorescent image in the lymphatic channel sheet (LCS) limb before the removal of the LCS (in 8th week) and after the removal of the LCS (in 12th week)

**FIGURE 8:**
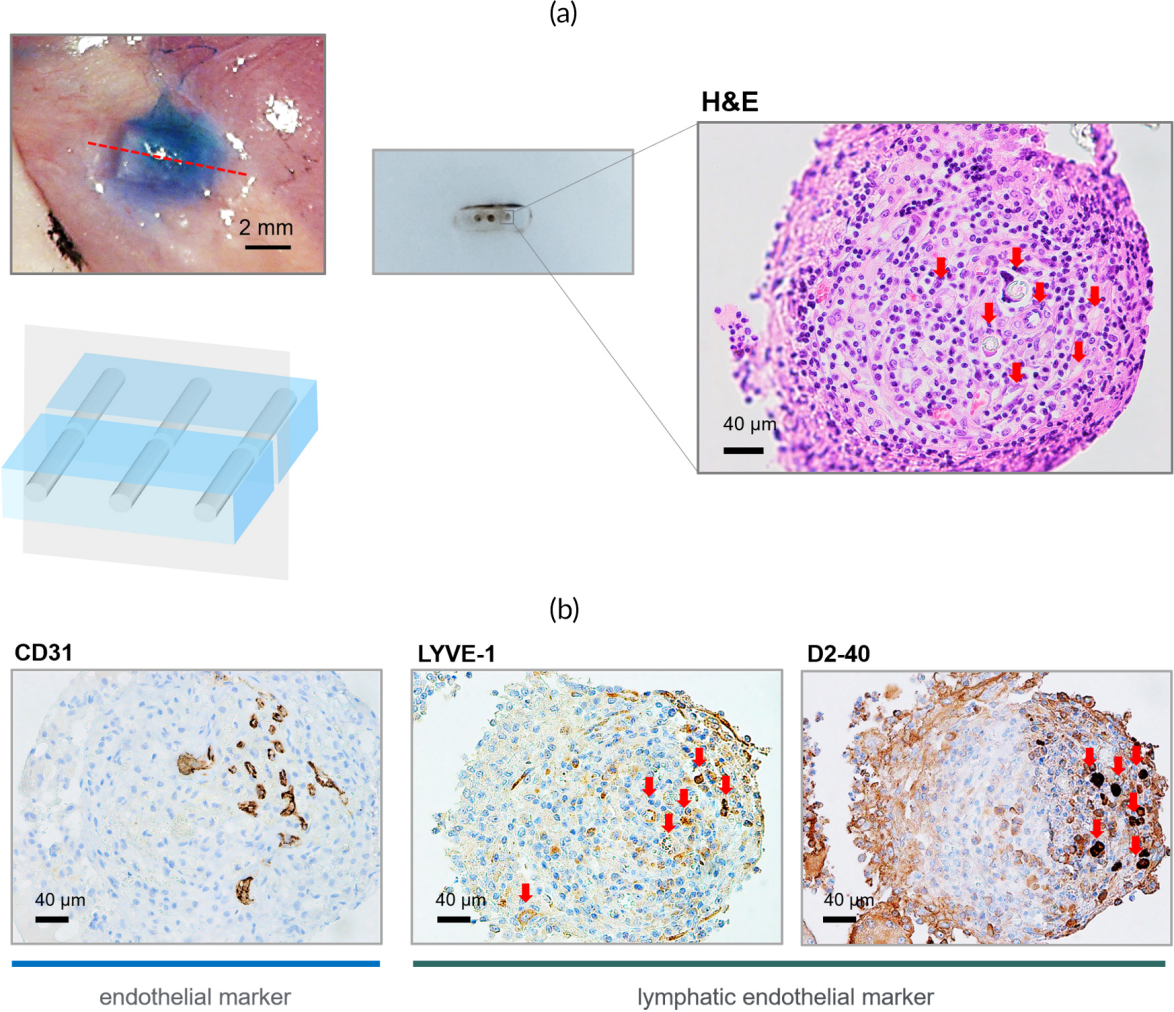
The histological analysis of the harvested lymphatic channel sheet (LCS) after 8 weeks of implantation. (a) The LCSs were cut perpendicular to the channel direction at the center and investigated the cross‐section of the channels with H&E staining. The red arrows present empty space inside the channels. (b) Immunohistochemistry analysis of endothelial cells for lymphatic and blood vascular regeneration inside the channels. There were several lymphatic vessels and blood vessels of 10–20 μm diameters inside the channels of the LCS.

After the follow‐up of 8 weeks, we harvested the LCS from each animal model. Removal of LCS caused an obstruction of lymph flow in the upper limb, and the linear pattern of ICG pattern was changed to a diffuse pattern around the lymphatic vessels and area where the LCS has been removed (Figure 7). It is verified that lymphatic flow was dependent on the pathway in the LCS. We also investigated lymphangiogenesis inside the channel of the LCS through the harvesting of the LCS. The harvested LCSs were fixed in paraffin blocks and the channel center was cut perpendicular to the channel direction to investigate the cross‐section of the channels. In the H&E staining section, most of the space was filled with cells, but several conduit structures existed (Figure 8a). Further evaluation with IHC staining distinguished endothelial cells using CD‐31, a pan‐endothelial marker, and lymphatics using LYVE‐1 and D2‐40, which are the markers of LECs. As shown in Figure 8b, several vessels of about 20 μm in diameter lined by endothelial cells existed inside the channel. Moreover, the lymphatic endothelium markers, LYVE‐1 and D2‐40, indicate that many lymphatic vessels of 10–20 μm grew inside the channels. Through this microscopic analysis, it was verified that multiple lymphatic vessels grew inside the channel, and lymph flow was maintained through these lymphatic vessels.

## 3 DISCUSSION

In cancer management, lymph node dissection is an indispensable procedure to check and inhibit cancer spread, and the ensuing absence or damage of lymph nodes provides a major cause of lymphedema because lymph nodes play an important role in fluid homeostasis.^36^, ^37^ The goal of this study is to develop a technique for implanting an artificial lymphatic structure, which is the LCS, that can replace the removed lymph nodes and maintain lymphatic flow. Although the role of the LCS was focused on the prevention of lymphedema, the treatment of implanting the artificial lymphatic structures was reported in 1908. Handley WS implanted the silk threads subcutaneously to drain lymphatic fluid in the affected arms of lymphedema patients.^38^ The silk threads presented only a short‐term effect due to infection, but it is meaningful because it was a first attempt to enhance lymphatic flow using artificial materials. After the silk threads, the artificial lymphatic structures have been developed continuously to rehabilitate lymphatic function (treatment) for patients or to prevent lymphatic dysfunction.

Some researchers have been trying to provide a pathway for edema fluid flow by subcutaneous implantation of silicone tubes in lymphedema patients.^39^, ^40^, ^41^, ^42^, ^43^ Olszewski et al. implanted the silicone tubes subcutaneously in 150 patients with lymphedema after pelvic and axillary lymphadenectomy and radiation therapy. This method intuitively aims to relieve lymphedema by providing a bypass for lymphatic flow for the treatment of lymphedema. The transport of lymphatic fluid was observed through the implanted silicone tube in clinical practice, and relief of edema was also confirmed over 2 years. Because silicone is a highly biocompatible and bio‐durable material, there were no significant inflammatory reactions in the implanted tube of limbs.^44^ Despite these results, the silicone tube had the disadvantage of being too bulky compared to the lymphatic structure and it requires a separate pump system for the movement of lymph fluid. Moreover, the silicone tube served as the lymphatic drainage pathway, but it could not play a role in inducing lymphangiogenesis. Recently, collagen fiber which is called Biobridge™ has been tried as a lymphatic scaffold by Hadamitzky et al. They evaluated the therapeutic efficacy of the nanofibrillar collagen scaffolds using animal models and patients.^45^, ^46^, ^47^ The scaffolds improved lymphatic drainage and lymphangiogenesis by combination with endothelial growth factors or stem cells. Because collagen is a biocompatible material that does not induce complications such as inflammation or infection, it is the most advanced lymphatic scaffold for both prevention and treatment of lymphedema. However, the problem of low treatment and prevention efficacy continues to be raised. Although microinterventional devices may be a major candidate technique for the treatment or prevention of lymphedema,^48^ the effective artificial lymphatic structure remains an unmet need in the medical field.

In an effective lymphatic structure, both the role of the lymphatic pathway and the scaffold for lymphangiogenesis are required to satisfy clinical unmet demands. The implanted LCS maintained lymphatic flow even after the dissection of BLN and radiation. The indicator, which is ALNs connected directly with BLNs, exhibited that the LCS connected lymphatic flow from the distal to the proximal area instead of BLN (Figure S2). All our diagnostic criteria of lymphedema including swelling, ICG patterns, and lymphatic drainage into proximal lymph nodes (ALNs) indicated that the lymphedema relief occurred in the LCS limbs in comparison with the resection limbs. Typical ICG patterns of lymphedema (splash, stardust, diffuse) were rarely observed in the LCS limbs, while they were frequently represented in the resection limbs. The results in volume measurement indicate that the distal area in the resection limb maintained relatively severe edema for 8 weeks and the volume change in the LCS limbs was not significant compared to normal. The LCS also served as a scaffold for lymphangiogenesis and vascularization. Multiple endothelium (blood vessels) and microscopic lymphatic vessels were identified inside the channel of the LCS by the histological analysis. Because the LCS was transplanted during lymph node dissection in this study, the role of the lymphatic pathway and scaffold was to prevent lymphedema. Meanwhile, the plastic surgeons who trained in microvascular lymphatic surgery have tried to perform LYmphatic Microsurgical Preventive Healing Approach (LYMPHA) technique to prevent lymphedema by lymph node dissection. Even though LYMPHA is an advanced microsurgical technique that creates a lymphatic‐venous bypass, it is still too difficult to be used in practice. Because the LCS is relatively easy to apply and was shown to effectively prevent lymphedema, it may be an effective alternative technique to prevent lymphedema after lymph node dissection.

An effective lymphatic structure requires good biocompatibility, chemical stability, flexibility, elasticity, and antiadhesion ability. Above all, the lymphatic structure should protect secured space for the pathway from fibrous encapsulation, which isolates it from the surrounding environment, because the isolation obstructs the pathway in maintaining lymphatic flow. The advantage of the LCS using PDMS material is the avoidance of adsorption and coagulation of blood and cells because of its hydrophobicity. We found fibrous tissue surrounding the LCS, but the fibrous tissue only blocked the leakage of lymphatic fluid between the LCS and lymphatic vessels and did not interrupt the lymphatic flow. The inside of the channels became a secured space not only for the lymphatic pathway but also for lymphangiogenesis. Because newly formed lymphatic vessels were very fine and weak, about tens of microns, they need to be protected from the surrounding environment to grow effectively. In addition, the LCS helped keep broken lymphatic vessels close enough for lymphangiogenesis to occur and reconnect lymphatic circulation. In the resection of limbs, shrinkage and retraction of broken lymphatic vessels occurred due to the removal of BLNs, which increased the distance of the cut ends of the lymphatic vessels from each other. Increasing the distance in which the lymphatics are absent decreases the possibility of lymphangiogenesis. The LCS procedure inhibited broken lymphatic vessels from becoming distant, increasing the possibility of lymphatic reconnection. The results of lymphatic reconnection in this study are considered to prove that the LCS of PDMS, which is a lymph‐friendly material, provided “a lymph‐friendly space” in the animal body.

Our study was limited in that the optimizing condition of the LCS was not characterized. Optimization of the physical properties and structure of the LCS is required in the next study for the application to clinical research. Although the animal model of secondary lymphedema seems to be appropriate for this research purpose, it was only the results in small animals. In the animals, edema and abnormal lymphatic drainage in ICG patterns were only maintained for 8 weeks, during the follow‐up period. However, the establishment of chronic lymphedema models was necessary to confirm the treatment effect of the LCS although we focused on the effect of prevention of lymphedema. The chronic lymphedema animal model allows for investigating whether the long‐term effect of LCS can be sustained. The consistency of other observations such as bioimpedance, MRI, or micro‐CT is also necessary to validate the effect of the macroscopic lymphatic flow, and quantification analysis using histological method may help to reveal the mechanism of lymphangiogenesis in the LCS.

## 4 CONCLUSION

We developed a technique to prevent secondary lymphedema using the two‐dimensional LCS based on PDMS material. The efficacy of the technique was evaluated in the animal model using several diagnostic methods. The LCS allowed the lymphatic flow to be maintained despite physical disconnection. and also served as a scaffold for lymphangiogenesis. Although further research is needed for clinical application, this study is the first experimental attempt of the sheet‐type microfluidic channel to prevent the disconnection of lymphatic flow in cancer treatment. These results have the potential to be utilized for the prevention and treatment of lymphedema in the clinical field.

## 5 MATERIALS AND METHODS

### 5.1 Study design of animal experiment

All animal procedures were reviewed and approved by the Ethics Committee of the Institutional Animal Care and Use Committee (IACUC) in the Asan Medical Center. The animals were kept in a light‐ and temperature‐controlled environment and permitted free access to water and standard laboratory chow. We used female Sprague–Dawley (SD) rats aged 8 weeks in the animal experiment (*n* = 13) because the female rats gained less weight and it is easy to identify ALNs on the skin by fluorescence dye. The experimental rats were separated into two groups; the nonsurgical observation group (*n* = 9) and the surgical observation group (*n* = 4). The nonsurgical observation group was used to obtain data without surgical methods such as volume measurement and ICG lymphangiography. On the other hand, we investigated lymphatic flow directly with visible‐light dye (EB dye) and near‐infrared fluorescent dye (ICG dye) in the surgical observation group. The total period of follow‐up was 8 weeks after surgery and radiation procedure and the LCSs were harvested on the 8th week to analyze histopathologically. The condition after removal of the LCS was also checked on the 12th week (Figure S3). Additional normal rats (*n* = 3) were used to compare the status of normal lymph flow with the experimental status.

We compared the difference between the resection limb, in which BLNs were removed completely, and the LCS limb, in which the LCS was implanted instead of the removed BLN, to verify its effects (Figure S4). The animal model in this study reduces errors due to differences between individuals by comparing both resection limb and LCS limb within one animal. The efficacy of the LCS was verified by visualizing the lymphatic pathway, measuring the volume of each limb, identifying the pattern of lymphatic drainage, and investigating the indicator lymph nodes. The “indicator lymph nodes” are ALNs that allow identifying of whether lymph flow was connected to the proximal area. The lymphatic fluid in the distal area is collected into BLNs and transferred to ALNs. If the lymphatic flow is normal, a lymphatic fluid including dye, which is injected at the distal area subcutaneously, will be transferred into the anatomical indicator that is the ALNs. Then, we can identify the reconnection of lymphatic flow by investigating the presence of the dye molecules (Figure S5).

### 5.2 Fabrication of PDMS microfluidic passage for LCS

Polydimethylsiloxane (PDMS) is a widely used polymer for fabrication materials such as biological microfluidic applications because it has nontoxicity, biocompatibility, and ease to produce fine microstructure.^49^ The process of fabrication for the LCS using a PDMS‐microfluidic sheet is shown in Figure S6a. The PDMS prepolymer (Sylgard 184, Dow Corning, Midland, MI, USA) was mixed with a curing agent in a 12:1 weight ratio and poured into the polystyrene Petri dishes. Mixed PDMS polymer was degassed with a vacuum pump at 0.09 MPa under vacuum and cured at room temperature. After 12 h (before curing is finished), the mold, which is made of the 32‐G syringe needles (outer diameter: 235 μm), was placed on the PDMS film and the PDMS polymer of the same mixing ratio was poured on top of the mold. The needle mold was removed after curing for 24 h in the vacuum chamber. The PDMS sheet was fabricated with 500–600 μm thickness and cut to include three to four passages of approximately 250 μm (Figure S6b). Before use, the PDMS‐based LCS was washed with acetone, methanol, and distilled water for 15 min each in a cleaning bath.

### 5.3 Surgical procedure

The SD rats were anesthetized with Tiletamine/Zolazepam (10 ml/kg; Zoletil, Virbac, France) mixed with Xylazine (volume ratio 5:1; Rumpun, Bayer Korea, Republic of Korea) after being induced by isoflurane gas in a concentration of 4% before the procedure. EB dye solution (30 μl of a 30 mg/ml solution in 0.9% saline; Sigma, MO, USA) was injected subcutaneously into the paws to visualize lymphatic vessels and lymph nodes after anesthesia. The collective lymphatic vessels usually are located on the dorsal side of the upper limbs and are connected to brachial lymph nodes (BLNs) directly. The BLNs are observed in the space between the lateral border of the anterior triceps brachii and the posterior latissimus dorsi. The efferent lymphatic vessel of the BLNs enters between the two muscles and connects to the ALNs. We removed subcutaneous fat surrounding the BLNs and exposed the lymph node, afferent lymphatic vessels, and efferent lymphatic vessels. The BLNs were dissected from both the resection limbs and the LCS limbs. In implantation of the LCS, its structure was fixed between one edge of tissue containing afferent lymphatic vessels and another edge of tissue containing efferent lymphatic vessels by using a polyglycolic acid braided synthetic absorbable suture in 8–0 size (AILEE, Republic of Korea). After the procedure, we made a proximal circumferential incision to the limbs and suture the folding skin from each side to disconnect the superficial lymphatics. The skin folding suture allows lymph flow was concentrated in the lymph node dissection area by blocking a potential pathway (Figure S4). All procedure was performed using a microsurgery system (F170; Carl Zeiss, Germany). Animals were maintained at a constant temperature of 20°C during the operation (Figure S7).

### 5.4 Inducing fibrosis by irradiation

Forty‐eight hours after surgery, rats were again anesthetized with low doses of Tiletamine/Zolazepam, and the surgical area of both limbs was irradiated using a single dose of 20 Gy delivered by a 320‐kVp X‐ray biological irradiator (X‐Rad320; Precision, CT, USA). The radiation was delivered in 10 fractions at a rate of 1 Gy/min to reduce damage to animals. Irradiation induces an inflammatory reaction, tissue fibrosis, and adhesion in the area performed by BLNs dissection and it is similar to the side effects of radiation therapy for cancer treatment (Figure S7). The rest of the body except for the limbs was protected by covering with three 2‐mm lead plates (totally 6‐mm thickness) which were customized for the animals.

### 5.5 ICG lymphangiography

ICG lymphangiography is a useful method for the evaluation of secondary lymphedema utilizing near‐infrared fluorescence dye as a tracer to monitor the direction, distribution, and dynamic change of lymphatic flow. The technique is safe, minimally invasive, and relatively easy to use. The change of ICG pattern is a well‐described diagnostic criterion in clinical secondary lymphedema of extremities. The lymphatic drainage pattern on ICG lymphography usually changes from a linear to a splash pattern, to stardust and then to a diffuse pattern as the severity of lymphedema progresses.^50^, ^51^, ^52^ We investigated lymphatic flow and indicator lymph nodes using a fluorescence dye (Indocyanine green, DID Indocyanine Green Inj, Dongindang Pharmaceutical Company, Republic of Korea) and a near‐infrared imaging system. The ICG fluorescence imaging system dedicated to the animal experiment was customized with a 730‐nm high‐power LED (LST1‐01G01‐FRD1‐00; Opulent Americas, NC, USA), bandpass filter (FF01‐832/27‐50‐D; Semrock, NY, USA), and a custom near‐infrared camera (Supp. Figure S8). The fluorescence images were observed for 30 min after injection of ICG dye solution (30 μl of a 1 mg/ml solution in bovine serum albumin solution of 2.5 mg/ml) in the paws (distal area). Prior to the imaging process, hair in the upper limb was removed with electric clippers and depilatory cream to prevent the disturbance of light.

### 5.6 Volume measurement

Swelling derived from the accumulation of lymphatic fluid is a typical sign of secondary lymphedema. Volume measurement of limbs is another criterion for the severity of lymphedema. Water displacement,^53^ circumference measurement,^54^ or measuring paw thickness^55^, ^56^ are commonly used for volume measurement in the preclinical animal model. However, most of them are developed for the hindlimb or tail model and have certain limitations. Because the upper limb of the rat is almost truncated in shape, the frustum approximation may be a more appropriate method to measure the volume of the upper limb in this study. We calculated the volume of each limb using the frustum approximation, which is expressed by

(1) $$V=\frac{1}{12}\pi \left({d_{c}}^{2}+d_{c}d_{e}+{d_{e}}^{2}\right)\sqrt{l^{2}-\frac{{\left(d_{c}-d_{e}\right)}^{2}}{4}}$$

where V is the volume of each limb, $d_{c}$ is the diameter of the carpal in the distal, $d_{e}$ is the diameter from elbow to cubital fossa, and l is the distance between the line of $d_{c}$ and $d_{e}$ (Figure S9). The diameter and the distance were measured by ImageJ software (ImageJ 1.53c, the National Institutes of Health, and the Laboratory for Optical and Computational Instrumentation, MD, USA) after obtaining images of the upper limbs at the same position. Each measurement of $d_{c}$, $d_{e}$, and l was performed three times by a single researcher to obtain the average values. We used a fixed stand for the camera and lighting device to increase measurement accuracy. The volume difference of LCS and resection limbs was compared by the paired Student's *t*‐test (*p* < 0.05) for the whole period.

### 5.7 Histological examination

The LCSs were harvested after 8 weeks of implantation and they were fixed with 4% formaldehyde. The fixed tissues were rinsed with tap water to remove the fixative for about 2 h. The LCSs were dehydrated in the graded ethanol and cleared in xylene using a tissue processor (Excelsior ES, Thermo Fisher Scientific, USA) and embedded into paraffin blocks sectionally using a paraffin embedding station (EG1150H; Leica, Germany). The paraffin blocks were cut into 5‐μm‐thick sections on a rotary microtome (RM2255; Leica, Germany). The paraffin sections were stained with hematoxylin and eosin (H&E) to identify the tissue structure inside the channels. For immunohistochemistry (IHC) staining of the sections, the following antibodies were used: rabbit polyclonal LYVE‐1 (1:100; NB100‐725, Novus Biologicals, CO, USA) as a lymphatic vessel marker, mouse monoclonal D2‐40 (1:100; ACR266B, Biocare Medical, CA, USA), which is a lymphatic endothelium marker, rabbit polyclonal CD31 (1:2000; ab182981, Abcam, UK), which is an endothelial cell marker. All histologic slides were acquired using a light microscope (Model BX40, Olympus, Japan).

### 5.8 Statistical analysis

The results of volume measurement are presented as the mean and standard deviation of the mean. The statistical analyses were performed using GraphPad Prism 9 (GraphPad Software Inc., CA, USA) and Microsoft Excel 2019 (version 2111, Microsoft Corporation, CA, USA). The *t*‐test and one‐way ANOVA were used to detect significant differences and a *p* value < 0.05 was considered to indicate a statistically significant difference in this study. The IQR presents statistical dispersion, which is the spread of the data. The values of the IQR were obtained by subtracting Q1 (the lower quartile, 25th percentile) from Q3 (the upper quartile, 75th percentile).

## AUTHOR CONTRIBUTIONS

**Hwayeong Cheon:** Data curation (lead); formal analysis (lead); funding acquisition (supporting); investigation (lead); methodology (lead); resources (lead); supervision (supporting); validation (equal); visualization (lead); writing – original draft (lead); writing – review and editing (equal). **Ma. Nessa Gelvosa:** Data curation (equal); formal analysis (equal); investigation (equal); methodology (equal); validation (equal); writing – original draft (supporting); writing – review and editing (equal). **Sang Ah Kim:** Data curation (equal); formal analysis (equal); investigation (equal); methodology (supporting); validation (equal); writing – review and editing (equal). **Ho‐Young Song:** Conceptualization (equal); project administration (supporting); resources (equal); validation (equal); writing – review and editing (equal). **Jae Yong Jeon:** Conceptualization (lead); data curation (equal); funding acquisition (lead); methodology (equal); project administration (lead); resources (lead); supervision (lead); validation (lead); writing – original draft (equal); writing – review and editing (lead).

## FUNDING INFORMATION

This work was supported by the National Research Foundation of Korea (NRF) grant funded by the Korea government (MSIT). (No. NRF‐2019R1A2C1009055, No. NRF‐2021R1F1A1056527).

### PEER REVIEW

The peer review history for this article is available at https://publons.com/publon/10.1002/btm2.10371.

## Supporting information


**Supplementary Figure 1** After implantation of the lymphatic channel sheet (LCS), investigation of the indicator (axillary lymph nodes, ALNs) in both the LCS and resection limb. L5 presented the result of poor lymph node dissection because lymph fluid was able to reach the proximal area beyond the surgical/irradiated area. L5 has been excluded from all measurements.
**Supplementary Figure 2** The comparison with lymphatic connection from distal to proximal are in (a) normal limb and (b) the LCS limb in which BLN was exchanged with the LCS.
**Supplementary Figure 3** Experimental design and schedule to verify the efficacy of the LCS in the animal models for secondary lymphedema.
**Supplementary Figure 4** The scheme of implantation of the LCS. The LCS (yellow triangle) was implanted between the pectoralis major and latissimus dorsi instead of the brachial lymph node. The lymphatic fluid flowed along the lymphatic vessels (LV) from the distal area to the ALNs of the proximal area. The superficial lymphatics of the distal skin including of implanted area were disconnected from the proximal area by the circumference‐folding suture.
**Supplementary Figure 5** The scheme for the indicator for verifying the effect of the LCS. Axillary lymph nodes (ALNs), which are anatomically connected to brachial lymph nodes (BLNs), were used as an indicator to investigate the reconnection of lymphatic flow in both the LCS limb and resection limb.
**Supplementary Figure 6** (a) The progress of fabricating the LCS using polydimethylsiloxane (PDMS). (b) The flow test inside the LCS which was included 3 ~ 4 channels using Evans Blue (EB) dye and indocyanine green (ICG) fluorescence dye.
**Supplementary Figure 7** The scheme for the surgical procedure of BLNs dissection and 20‐Gy radiation. After the lymph node dissection and radiation, the LCS was implanted instead of BLNs in the LCS limb while no implantation was performed in the resection limb.
**Supplementary Figure 8** The customized near‐infrared imaging system for ICG lymphangiography in this study.
**Supplementary Figure 9** The calculation method how to obtain the formula 1. The frustum approximation was used for volume measurement of the rat upper limbs in this study.

## Data Availability

The data in this study are available from the corresponding author upon reasonable request.
