# Supplementary material for: Lymphatic channel sheet of polydimethylsiloxane for preventing secondary lymphedema in the rat upper limb model
Source: Bioeng Transl Med. 2022 Jul 5;8(1):e10371. doi: 10.1002/btm2.10371 (PMC9842043; doi:10.1002/btm2.10371)
Supplement: Supplementary file 1 — Supplementary Figure 1 After implantation of the lymphatic channel sheet (LCS), investigation of the indicator (axillary lymph nodes, ALNs) in both the LCS and resection limb. L5 presented the result of poor lymph node dissection because lymph fluid was able to reach the proximal area beyond the surgical/irradiated area. L5 has been excluded from all measurements. Supplementary Figure 2 The comparison with lymphatic connection from distal to proximal are in (a) normal limb and (b) the LCS limb in which BLN was exchanged with the LCS. Supplementary Figure 3 Experimental design and schedule to verify the efficacy of the LCS in the animal models for secondary lymphedema. Supplementary Figure 4 The scheme of implantation of the LCS. The LCS (yellow triangle) was implanted between the pectoralis major and latissimus dorsi instead of the brachial lymph node. The lymphatic fluid flowed along the lymphatic vessels (LV) from the distal area to the ALNs of the proximal area. The superficial lymphatics of the distal skin including of implanted area were disconnected from the proximal area by the circumference‐folding suture. Supplementary Figure 5 The scheme for the indicator for verifying the effect of the LCS. Axillary lymph nodes (ALNs), which are anatomically connected to brachial lymph nodes (BLNs), were used as an indicator to investigate the reconnection of lymphatic flow in both the LCS limb and resection limb. Supplementary Figure 6 (a) The progress of fabricating the LCS using polydimethylsiloxane (PDMS). (b) The flow test inside the LCS which was included 3 ~ 4 channels using Evans Blue (EB) dye and indocyanine green (ICG) fluorescence dye. Supplementary Figure 7 The scheme for the surgical procedure of BLNs dissection and 20‐Gy radiation. After the lymph node dissection and radiation, the LCS was implanted instead of BLNs in the LCS limb while no implantation was performed in the resection limb. Supplementary Figure 8 The customized near‐infrared imaging system for [file BTM2-8-e10371-s001.docx]

Lymphatic channel sheet of polydimethylsiloxane for preventing secondary lymphedema in the rat upper limb model

Hwayeong Cheon^1^, Ma Nessa Gelvosa^2^, Sang-Ah Kim^2^, Ho-Young Song^3^, and Jae Yong Jeon^2^*

^1^ Biomedical Engineering Research Center, Asan Institute for Life Sciences, Asan Medical Center, Seoul, Republic of Korea

^2^ Department of Rehabilitation Medicine, Asan Medical Center, University of Ulsan College of Medicine, Seoul, Republic of Korea

^3^ Department of Minimal-Invasive Intervention, The Affiliated Cancer Hospital of Zhengzhou University, Zhengzhou City, China

*Corresponding Author: jyjeon71@gmail.com

**Supplementary materials**


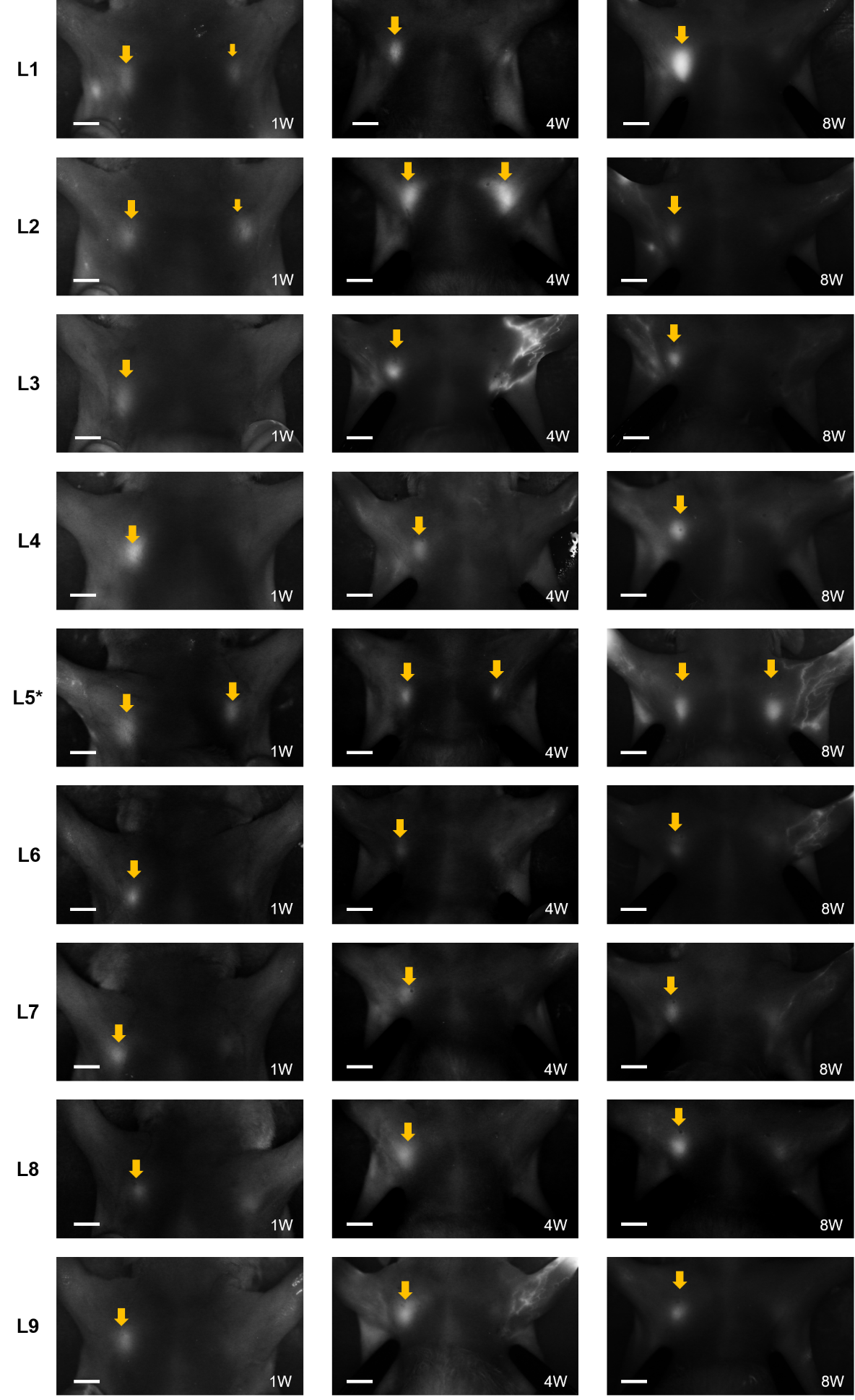


**Supplementary Figure 1** After implantation of the lymphatic channel sheet (LCS), investigation of the indicator (axillary lymph nodes, ALNs) in both the LCS and resection limb. L5 presented the result of poor lymph node dissection because lymph fluid was able to reach the proximal area beyond the surgical/irradiated area. L5 has been excluded from all measurements.


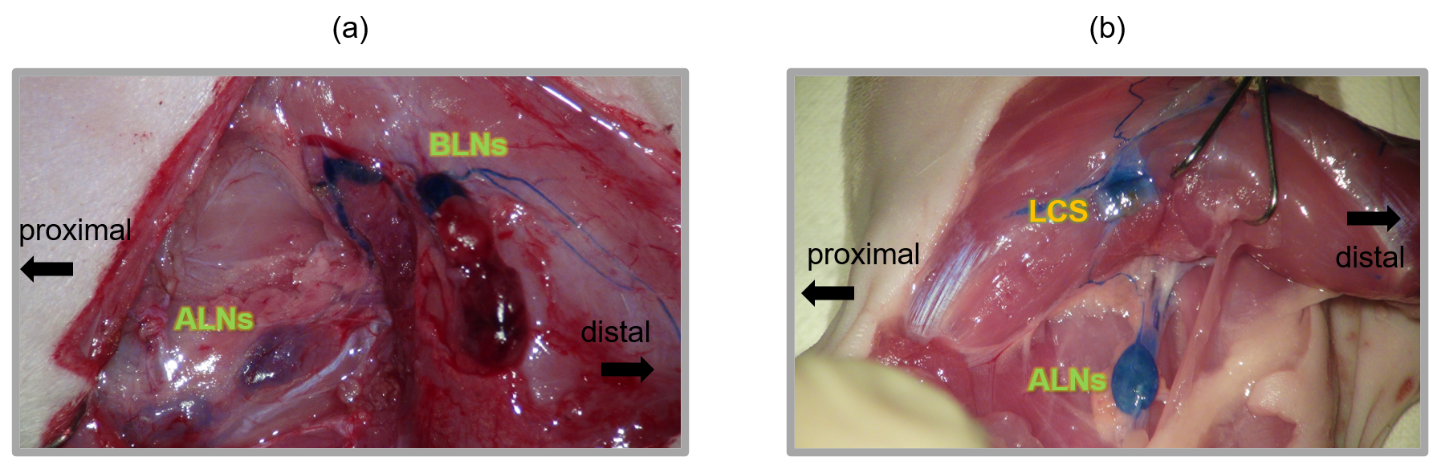


**Supplementary Figure 2** The comparison with lymphatic connection from distal to proximal are in (a) normal limb and (b) the LCS limb in which BLN was exchanged with the LCS.

**
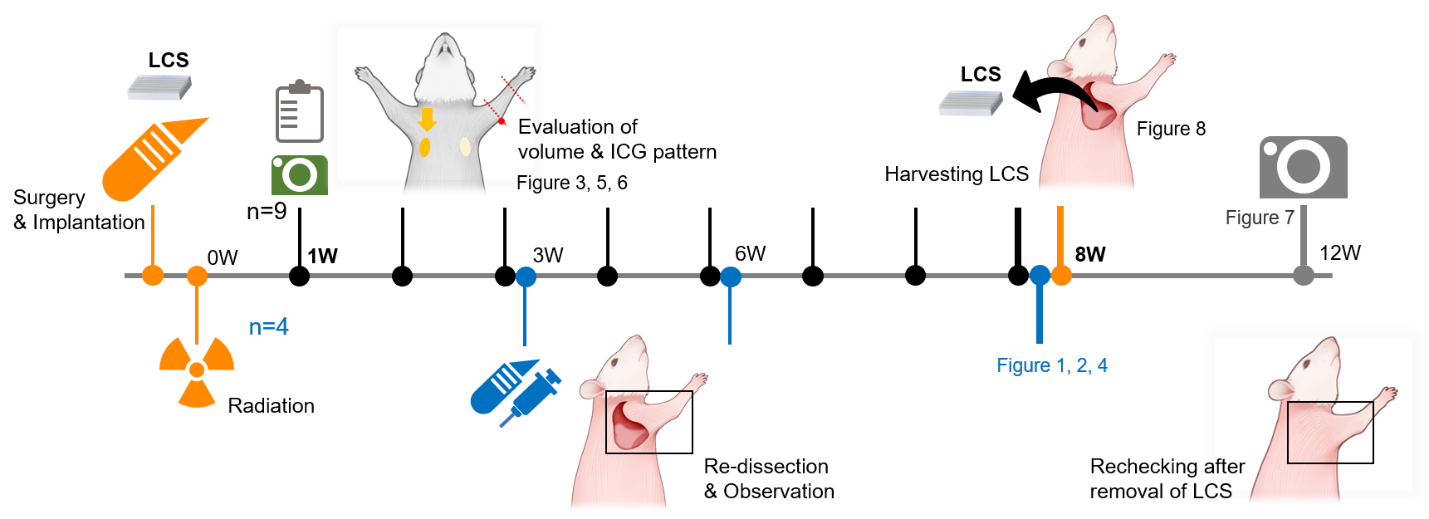
**

**Supplementary Figure 3** Experimental design and schedule to verify the efficacy of the LCS in the animal models for secondary lymphedema.

**
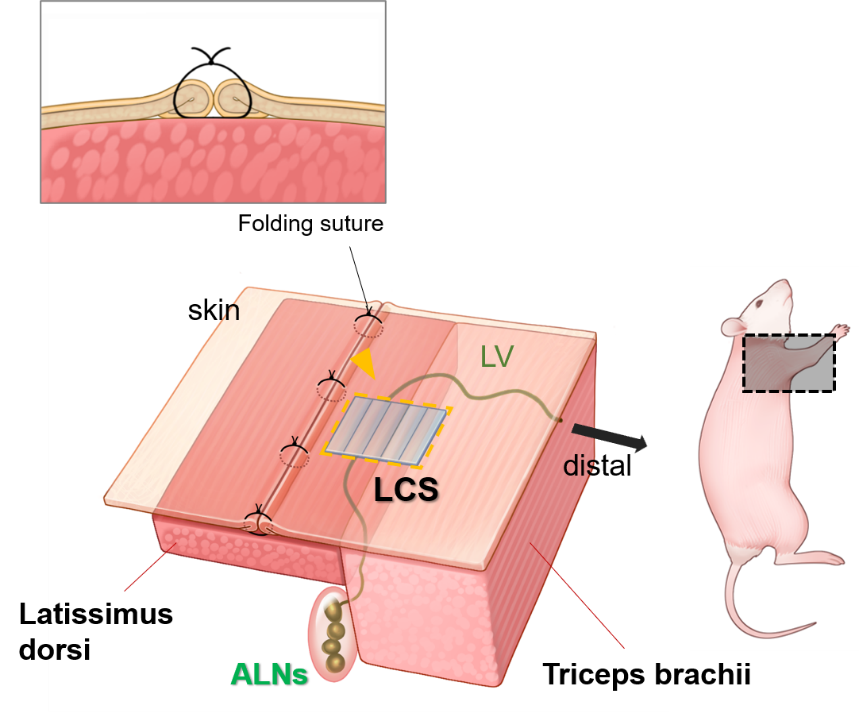
**

**Supplementary Figure 4** The scheme of implantation of the LCS. The LCS (yellow triangle) was implanted between the pectoralis major and latissimus dorsi instead of the brachial lymph node. The lymphatic fluid flowed along the lymphatic vessels (LV) from the distal area to the ALNs of the proximal area. The superficial lymphatics of the distal skin including of implanted area were disconnected from the proximal area by the circumference-folding suture.


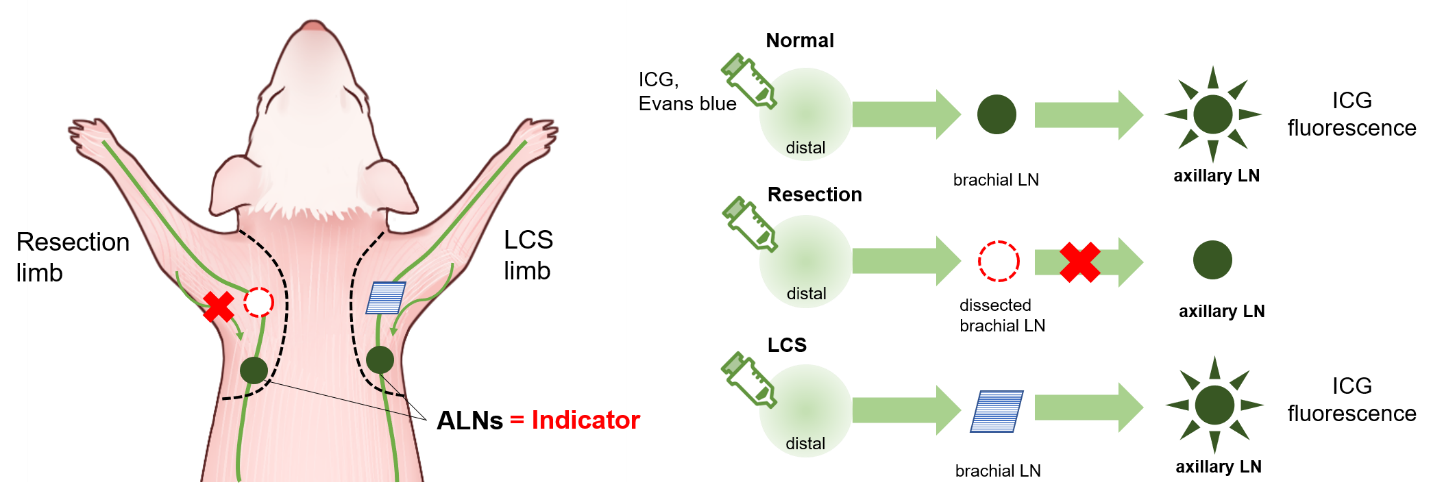


**Supplementary Figure 5** The scheme for the indicator for verifying the effect of the LCS. Axillary lymph nodes (ALNs), which are anatomically connected to brachial lymph nodes (BLNs), were used as an indicator to investigate the reconnection of lymphatic flow in both the LCS limb and resection limb.


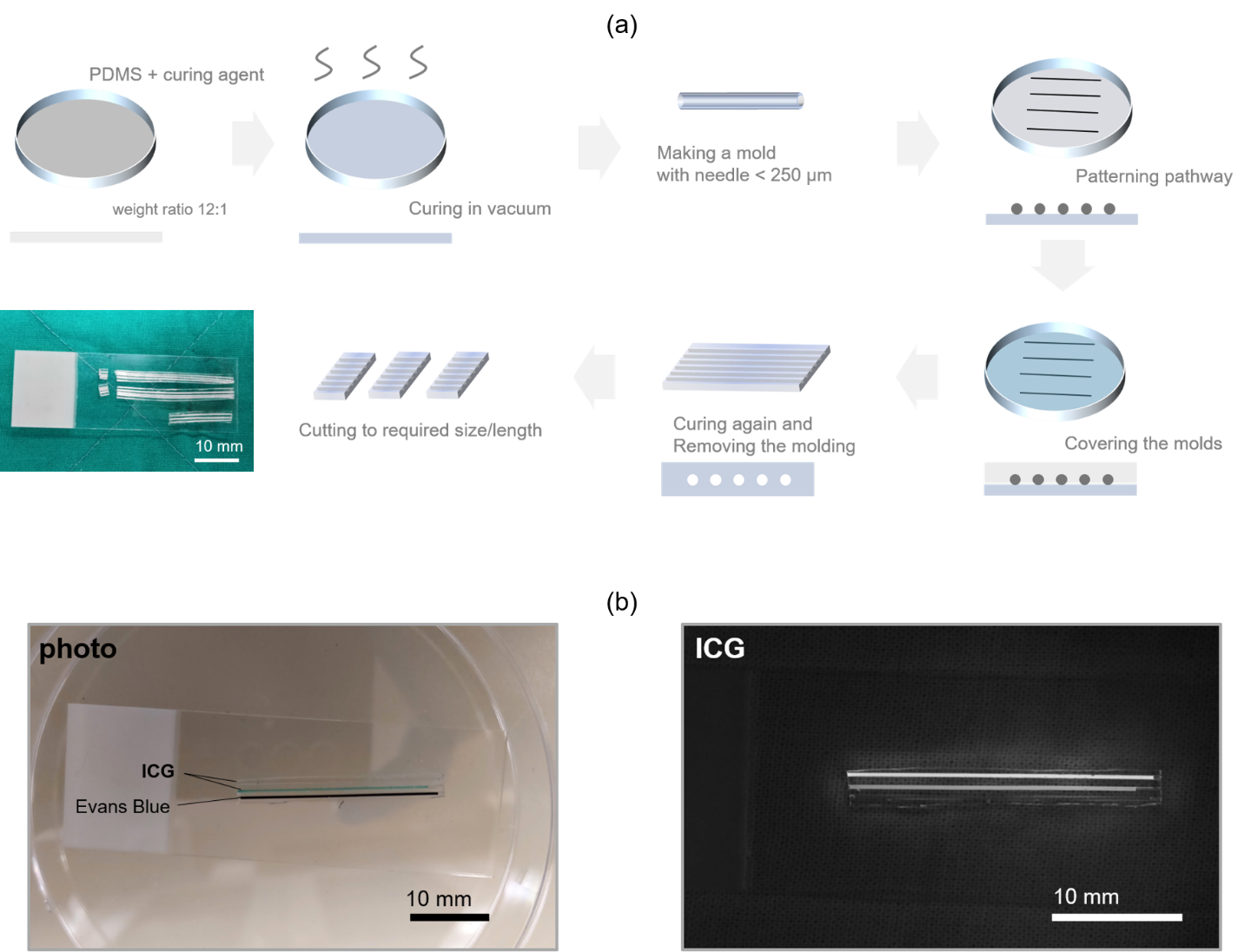


**Supplementary Figure 6** (a) The progress of fabricating the LCS using polydimethylsiloxane (PDMS). (b) The flow test inside the LCS which was included 3~4 channels using Evans Blue (EB) dye and indocyanine green (ICG) fluorescence dye.


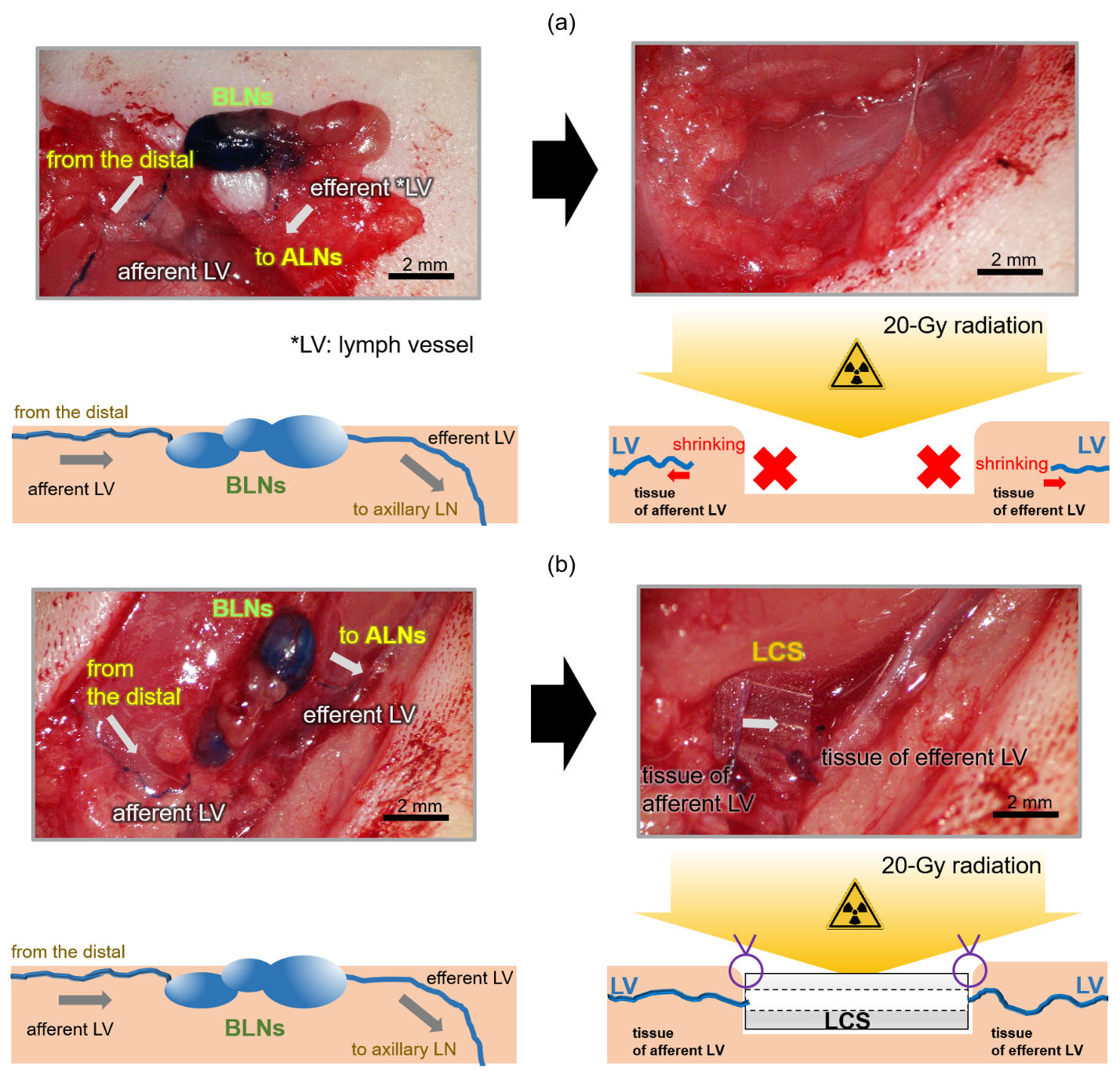


**Supplementary Figure 7** The scheme for the surgical procedure of BLNs dissection and 20-Gy radiation. After the lymph node dissection and radiation, the LCS was implanted instead of BLNs in the LCS limb while no implantation was performed in the resection limb.


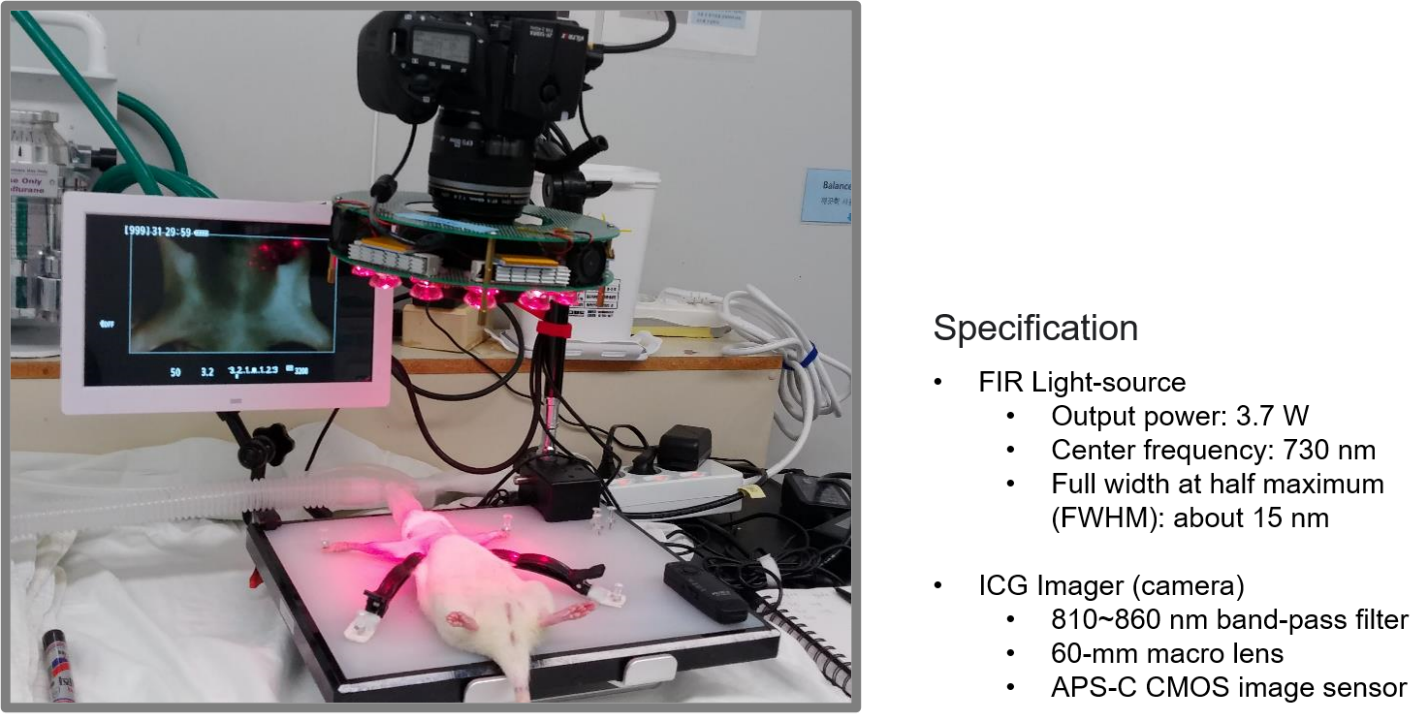


**Supplementary Figure 8** The customized near-infrared imaging system for ICG lymphangiography in this study.


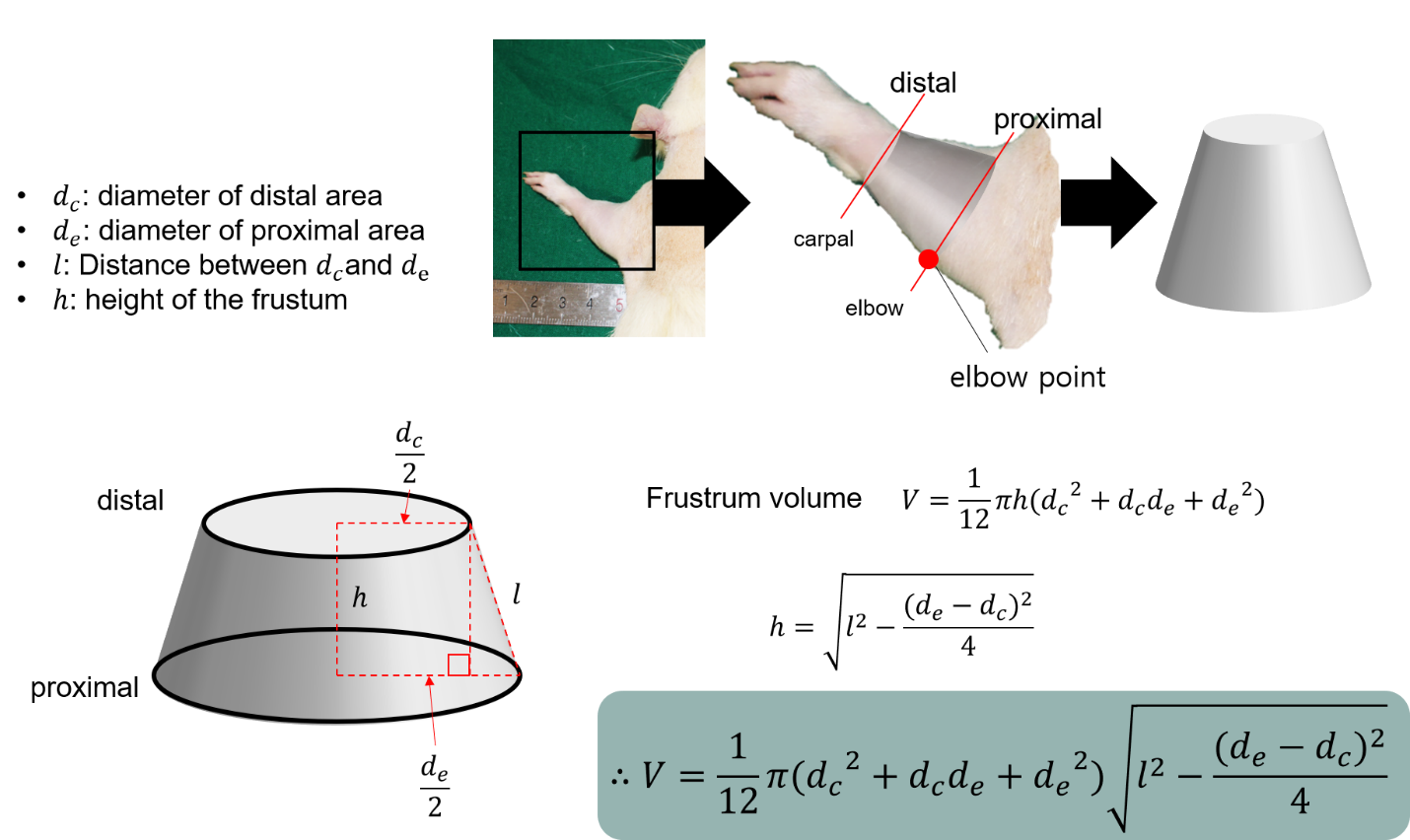


**Supplementary Figure 9** The calculation method how to obtain the formula 1. The frustum approximation was used for volume measurement of the rat upper limbs in this study.
